# Endoplasmic Reticulum Stress, Unfolded Protein Response, and Cancer Cell Fate

**DOI:** 10.3389/fonc.2017.00078

**Published:** 2017-04-26

**Authors:** Marco Corazzari, Mara Gagliardi, Gian Maria Fimia, Mauro Piacentini

**Affiliations:** ^1^Department of Health Sciences, University of Piemonte Orientale “A. Avogadro”, Novara, Italy; ^2^Department Clinical Epidemiology and Translational Research, INMI-IRCCS “L. Spallanzani”, Rome, Italy; ^3^Department of Biology, University of Rome “Tor Vergata”, Rome, Italy; ^4^Department of Biological and Environmental Sciences and Technologies (DiSTeBA), University of Salento, Lecce, Italy

**Keywords:** endoplasmic reticulum stress, unfolded protein response, E2F, autophagy, BRAF

## Abstract

Perturbation of endoplasmic reticulum (ER) homeostasis results in a stress condition termed “ER stress” determining the activation of a finely regulated program defined as unfolded protein response (UPR) and whose primary aim is to restore this organelle’s physiological activity. Several physiological and pathological stimuli deregulate normal ER activity causing UPR activation, such as hypoxia, glucose shortage, genome instability, and cytotoxic compounds administration. Some of these stimuli are frequently observed during uncontrolled proliferation of transformed cells, resulting in tumor core formation and stage progression. Therefore, it is not surprising that ER stress is usually induced during solid tumor development and stage progression, becoming an hallmark of such malignancies. Several UPR components are in fact deregulated in different tumor types, and accumulating data indicate their active involvement in tumor development/progression. However, although the UPR program is primarily a pro-survival process, sustained and/or prolonged stress may result in cell death induction. Therefore, understanding the mechanism(s) regulating the cell survival/death decision under ER stress condition may be crucial in order to specifically target tumor cells and possibly circumvent or overcome tumor resistance to therapies. In this review, we discuss the role played by the UPR program in tumor initiation, progression and resistance to therapy, highlighting the recent advances that have improved our understanding of the molecular mechanisms that regulate the survival/death switch.

## Introduction

The endoplasmic reticulum (hereafter ER) is the widest intracellular organelle spanning from the nuclear envelope to the cell membrane. It is immediately adjacent to the cell nucleus; its membrane is continuous with the outer membrane of the nuclear envelope and is characterized by an extremely expanded membrane delimiting an intra-organelle space named ER lumen. The extension/size of this organelle depends of cell’s activity and type (discussed later) and is organized in subdomains of different shapes such as tubules and cisternae, giving rise to two main dynamic and interconvertible structures: the smooth endoplasmic reticulum (SER) and the rough endoplasmic reticulum (RER), with membranes of the latter being decorated by ribosomes transiently attached to the external side (1). The ER is deputed to several different activities, including calcium storage, detoxification of chemical compounds, and lipid synthesis. It is also responsible for the correct folding and posttranslational modification of proteins destined to other organelles, the plasma membrane, as well as the extracellular compartment. While this activity resides in the RER, lipids to be delivered to other intracellular organelles are synthesized in the SER. The ER also represents the most important storage for intracellular calcium ions (Ca^2+^), this being required for the physiological activities of the compartment contributing to sustain the correct oxidoreductase potential. In fact, compared to the cytosol, the ER has a greater calcium concentration, conferring a more oxidizing redox potential on this organelle. Enzymatic modification of newly synthesized polypeptides, including disulfide bridge formation and carbohydrate addition, depends upon the maintenance of sufficiently oxidizing conditions within the ER lumen. An extremely reducing ER environment is unfavorable to disulfide bond formation, whereas an overly oxidizing ER may result in the trapping of proteins in a misfolded state (2).

**Figure 1:**
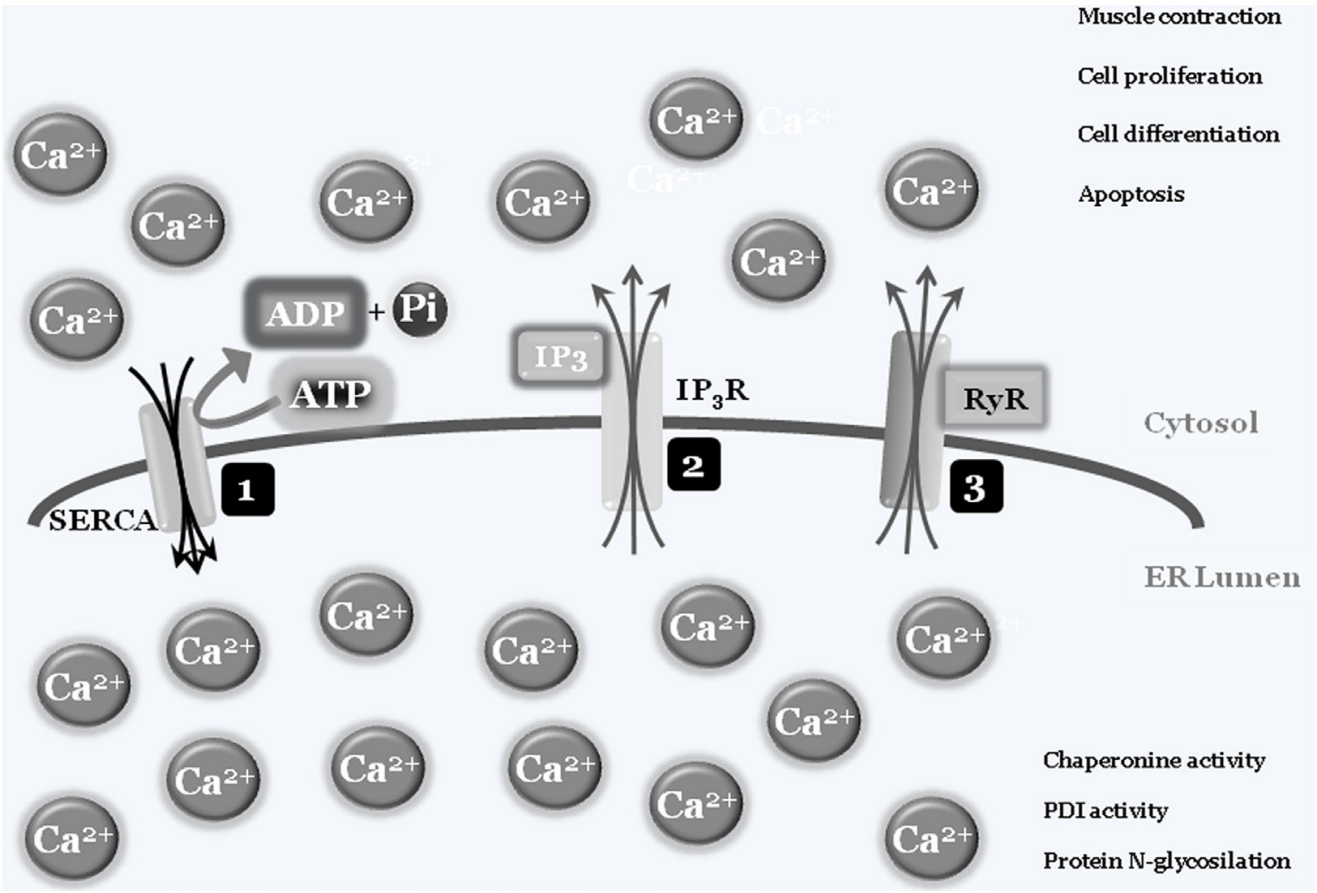
**Endoplasmic reticulum (ER)/cytosolic calcium exchange**. Physiologic low cytoplasmic calcium concentration is the result of SERCA pump activity that transfers free cytosolic calcium ions into ER lumen, using ATP molecules as a fuel source. The adequate ER calcium concentration establishes the luminal redox potential required for chaperons, protein disulfide-isomerases (PDIs), protein N-glycosylation, and more activities (1). Under specific stimuli, such as electric and/or IP3 production, transmembrane ER protein channels [IP3 receptor (IP_3_R) (2) and ryanodines (RyRs) (3)] will open and calcium ions will spread into the cytosol. The increased cytosolic calcium concentration will be used by cells to drive several activities such as contraction, proliferation, differentiation, and cell death. To block or inhibit these activities, the combined closure of IP_3_R and RyRs channels together with SERCA activity will lower cytosolic calcium concentrations to the physiologic level.

In some specialized cells and tissues such as muscles, “calcium storage” represents the organelle’s main activity, with the ER’s structure spanning the cells. Briefly, intracellular calcium is normally captured by specialized ATP-consuming pumps, the SERCA pumps (sarco/endoplasmic reticulum Ca^2+^-ATPase), to limit the cytosolic calcium concentration, thus avoiding abnormal and deleterious enzyme activation, such as caspases and calpains, or interaction with modulatory molecule such as calmodulins (3). Entrapped calcium can be released at convenience, by electrical signals or by secondary messengers binding to specific ER-transmembrane ions channels such as ryanodine and IP3 receptors, respectively. Once released into the cytosol, calcium ions can activate specific enzymes, to mediate specific cell responses such as differentiation and/or cell death, or resulting in specific activity such as muscle contraction. To shut down the signaling, calcium is then actively re-captured by SERCA *via* an ATP-dependent event, to re-establish the physiological low cytosolic calcium concentration, therefore closing the circle. As mentioned earlier, the correct ER luminal calcium concentration is also a fundamental requirement for the protein folding and posttranslational modification activities of this organelle, since chaperonins, protein disulfide isomerases (PDIs), N-glycosylating, and other enzymes all require the correct oxidoreductase potential to work/function properly (4, 5) (Figure 1).

## ER Stress Induction and Unfolded Protein Response (UPR)

When the ER protein folding capacity is overwhelmed, cells undergo a condition defined as ER stress, characterized by misfolded proteins accumulated inside the ER lumen. Several conditions can compromise the homeostasis of this compartment, so inducing an ER stress status, such as nutrient deprivation, hypoxia, and calcium depletion. To overcome the imbalanced ER protein-folding capacity, cells have evolved an evolutionary conserved signal transduction pathway called UPR and whose primary aim is to re-establish ER homeostasis (6). The UPR is controlled by three ER-transmembrane stress sensors, namely inositol-requiring enzyme 1α (IRE1α) (7), pancreatic endoplasmic reticulum kinase (PERK) (8), and activating transcription factor 6 (ATF6) (9, 10). Under physiological conditions, the activation of these sensors is inhibited by the binding of their luminal domains with the main and most represented ER-resident chaperone BIP/GRP78 (78-kDa glucose-regulated protein). In fact, BIP establishes a dynamic equilibrium between unfolded proteins (to be folded) and intra-luminal domains of the three ER stress sensors. Accumulated unfolded proteins determine an impaired equilibrium leading to BIP dissociation from ER stress sensors to massively cooperate in protein folding (due to its higher natural affinity to unfolded proteins compared to ER stress sensor luminal domains). The consequences of this disequilibrium are the homodimerization of both IRE1 and PERK, their trans-auto-phosphorylation and activation, paralleled by ATF6 translocation to the Golgi apparatus and subsequent activation (11, 12).

### Pancreatic Endoplasmic Reticulum Kinase

Pancreatic endoplasmic reticulum kinase is a type I transmembrane protein with a cytosolic serine/threonine kinase domain, highly present at mitochondria-associated ER membranes. Active PERK phosphorylates eukaryotic initiation translation factor 2α (eIF2α) causing a temporary inhibition of cap dependent while increasing the cap-independent translation of many mRNAs, such as activating transcription factor 4 (ATF4). ATF4, in turn, favors the expression of antioxidant response, amino acid biosynthesis, and transport genes to sustain cell survival. ATF4 also promotes the expression of growth arrest and DNA damage-inducible protein 34 which upon interaction with PP1A dephosphorylates eIF2α (to restore normal translation) and contributes to the late expression of C/EBP homologous protein (CHOP) to mediate ER stress-induced apoptosis. Therefore, depending on the severity and duration of stress, PERK activation can lead to either survival or cell death (13–15).

### Inositol-Requiring Enzyme 1α

Similarly, IRE1α is a type I transmembrane protein with a cytosolic serine/threonine kinase domain. Its activation triggers a kinase activity and an endoribonuclease activity promoting an atypical splicing of X-box-binding protein (XBP1) mRNA, localized nearby the ER membrane, to form a transcriptionally active mRNA, named XBP1s (spliced). In fact, un-slipiced XBP1 is usually non- or poorly translated, depending on cell type, while XBP1s is actively converted into the transcription factor XBP1, able to enter the nucleus to regulate the expression of genes taking part to protein folding, trafficking, and ER-associated protein degradation program (ERAD) processes. In addition, XBP1s promote cell survival since it inhibits CHOP expression and regulates the expression of genes involved in secretory pathways and in the expansion of ER compartment. On the other hand, activated IRE1α is also able to bind TNF receptor-associated factor 2 (TRAF2) which in turn engages apoptosis signal-regulating kinase 1 and JUN N-terminal kinase (JNK), leading to the activation of pro-apoptotic BIM and inhibiting anti-apoptotic BCL-2. In addition, the ribonuclease activity of IRE1 is able to selectively cleave ER-targeted mRNAs, thus contributing to protein income inhibition (16, 17).

### Activating Transcription Factor 6

**Figure 2:**
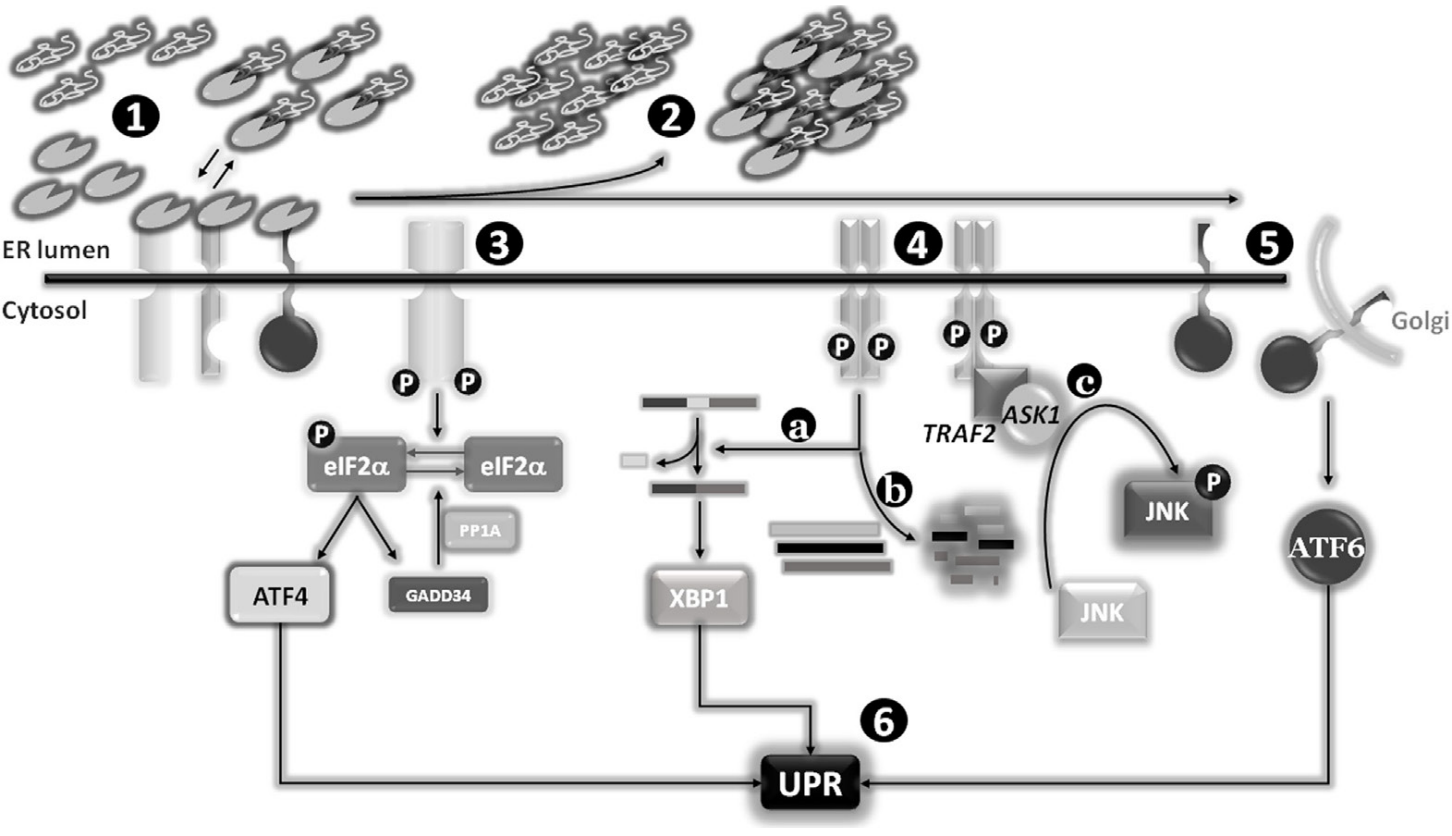
**Endoplasmic reticulum (ER) stress sensors, unfolded protein response (UPR) activation, and signaling pathways**. Under physiological conditions, the expression levels of the main ER chaperone Bip are sufficient to establish a dynamic equilibrium between the pool of newly synthesized proteins to be folded and the binding to luminal domains of the three ER stress sensors pancreatic endoplasmic reticulum kinase (PERK), IRE1, and activating transcription factor 6 (ATF6). The latter interactions are required to maintain these transmembrane proteins in a monomeric inactive status (1). Stimuli responsible for an increase of or an accumulation of unfolded or misfolded proteins into the ER lumen will determine a shift of the “Bip equilibrium,” resulting in the dissociation of this factor from the three ER stress sensors to increase the levels of “free” Bip to use as chaperone (2). The dissociation of Bip from these sensors will determine their activation: PERK and IRE1 will form omo-dimers/multimers which after trans-phosphorylation will become active (3) and (4), whereas ATF6 will reach the Golgi apparatus were two proteases (SP1 and SP2) will release the cytosolic and active transcription factor (5). The activation of the three sensors will drive the “UPR” program (6). Besides the splicing of X-box-binding protein (XBP1) as part of the classical UPR (4a), IRE1 is also able to specifically degrade several mRNAs (coding for pro-apoptotic factors?) (4b), and activate the stress protein JUN N-terminal kinase (JNK) through the recruitment of TNF receptor-associated factor 2 (TRAF2) and apoptosis signal-regulating kinase 1 (ASK1) (4c).

Activating transcription factor 6 is a type II transmembrane protein characterized by a cAMP-responsive element-binding protein/ATF basic leucine zipper domain. Upon ER stress induction, the BIP dissociation drives its translocation to the Golgi apparatus where it is cleaved by S1P and S2P proteases, generating a cytosolic active transcription factor. There are two known ATF6 homologs: (i) ATF6α, which regulates the expression of genes involved in the ER capacity and the expression of XBP1, and (ii) ATF6β, which inhibits the activities of the α isoform (9, 10) (Figure 2).

The ERAD system represents a complex and finely regulated pathway, whereby proteins are linearized, retro-translocated into the cytosol, ubiquitinated, and directed to proteasome to be degraded. It represents an efficient and fast strategy developed by cells to decrease the ER lumen overflow by unfolded, misfolded, or damaged proteins. Since this pathway is not this dissertation’s main topic, it will be not described further [for more details, we suggest the following publications (18, 19)].

## To Die or not to Die: The Key Role of E2F1

**Figure 3:**
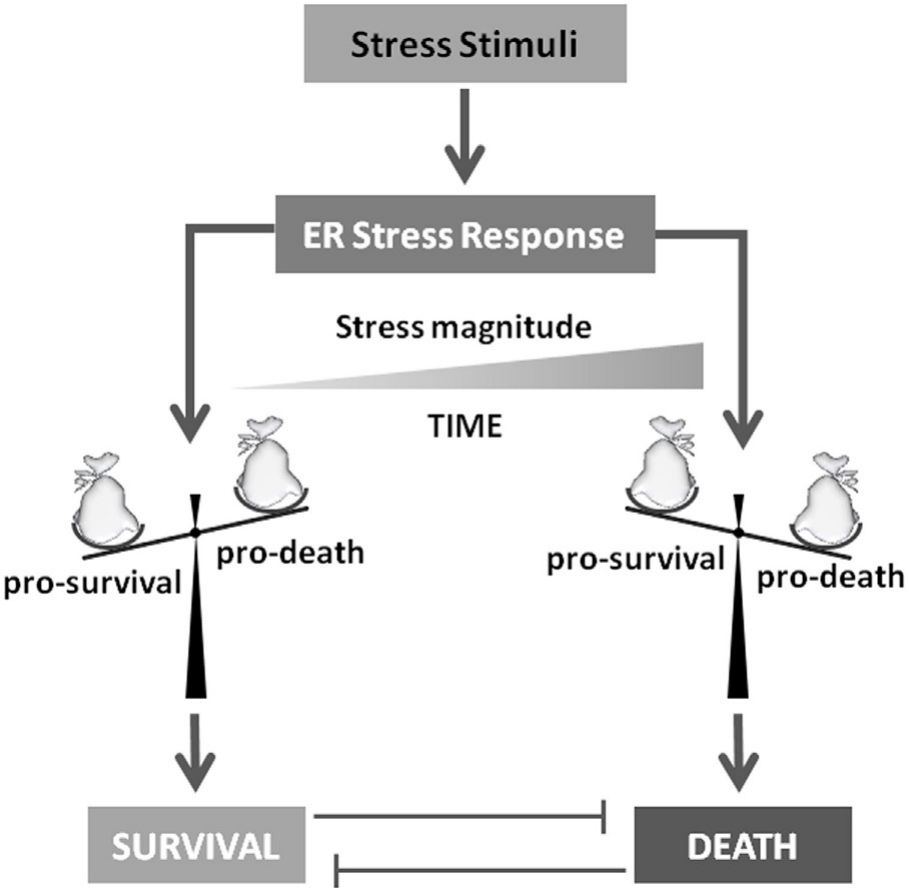
**To die or not to die**. Stress stimuli responsible for unfolded protein response activation will determine cell survival or death induction. Several evidences indicate that this crucial decision mainly depends on stress magnitude and duration. In fact, these players will determine the accumulation of pro-survival or pro-death factors that will drive the final cell decision.

**Figure 4:**
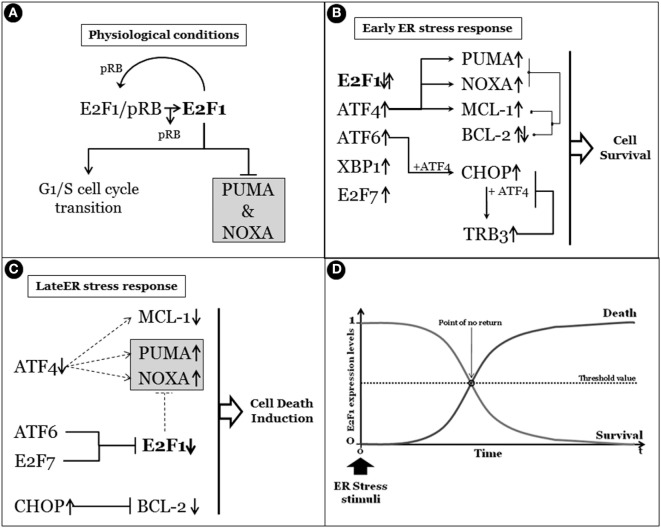
**Survival/death cell decision, a matter of E2F1**. **(A)** Under physiological conditions, E2F1 is in a dynamic equilibrium between the free form and the binding with pRb. Importantly, while the pRb binding inhibits its activity, free E2F1 regulates the G1–S cell cycle transition and the basal expression of pro-apoptotic PUMA and NOXA. **(B)** The early endoplasmic reticulum (ER) stress response determines the upregulation and/or activation of the unfolded protein response early genes/factors such as activating transcription factor 4 (ATF4), X-box-binding protein, activating transcription factor 6 (ATF6), and also E2F7, while E2F1 expression levels are not influenced. Importantly, although the expression of PUMA and NOXA are enhanced by ATF4, their pro-apoptotic activity is efficiently counteracted by the concomitant expression of anti-apoptotic factors such as MCL-1 (upregulated by ATF4) and BCL-2. Moreover, the pro-apoptotic activity of C/EBP homologous protein (CHOP) (GADD153), whose expression is enhanced by both ATF4 and ATF6, is also inhibited by downstream TRB3. In fact, TRB3 is a target of CHOP/ATF4 that blocks the CHOP and ATF4 function by binding to them, in the early stages of ER stress response. **(C)** Prolonged stress conditions determine the switch between the survival/death ER response characterized by ATF4 expression decline and subsequent reduction of target genes such as MCL-1, PUMA, and NOXA. Moreover, prolonged CHOP activation also results in BCL-2 expression inhibition. However, a gradual active decrease of E2F1 expression levels occurs under sustained ER stress due to ATF6 and E2F7 activities, resulting in removal of E2F1-dependent basal expression inhibition of both PUMA and NOXA that will induce the apoptotic program. In panel **(D)**, a schematic representation of a time-dependent ER stress result is reported, in which the correlation between the expression levels of E2F1 and the survival/death cell’s outcome under ER stress conditions is highlighted.

As described earlier, UPR outcomes are (i) generalized inhibition of protein synthesis, (ii) increased protein folding/posttranslational modification capacities of ER, and (iii) degradation of unfolded/misfolded or damaged proteins by the ERAD system, collectively representing pro-survival activities to sustain cell life. However, under prolonged or sustained ER stress conditions, the UPR program might fail to restore ER homeostasis and a UPR-mediated cell death program is therefore induced (20, 21). A key question is consequently: “How do cells discern between life or death decision under ER stress conditions?” A generalized and widely accepted hypothesis is that upon stress both pro-survival and pro-death factors are concomitantly transduced. However, the former is the most represented, at least initially, thus sustaining cell survival, efficiently inhibiting the activity of the latters. On the other hand, prolonged or sustained stress allows the accumulation of pro-death players, which in turn inactivate or inhibit the activity of pro-survival factors, resulting in cell death induction, mainly though the IRE1 pathway (22) (Figure 3). In fact, the aforementioned IRE1 activities, namely, (i) XBP1 mRNA splicing, (ii) regulated IRE1-dependent decay of mRNAs and JNK/p38 activation, seem to be responsible for the life/death switch under prolonged ER stress conditions (16, 23) (Figure 2). However, again, although the mechanism of cell death prevention or apoptosis induction have been deepened at molecular level, the nature and function of the real “switch” or “rheostat” remain elusive. Recently, the role of E2F1 has been described as highlighting a potential mechanistic survival/death switch under ER stress conditions (24). E2F1 is a member of the E2F family of transcription factors involved in several cellular functions such as proliferation, differentiation, and cell death (25, 26). In this model, the time-related selective downregulation of E2F1 expression is critically involved and required to switch between survival and death cell decision under ER stimuli (24). In fact, upon ER stress induction, the typical and well-consolidated UPR program is activated, with early activation of IRE1 and consequent unconventional splicing of XBP1 mRNA to produce the active transcription factor. The latter, then, will positively regulate its target genes deputed to increase ER-folding capacities and to setup the ERAD system. However, among those XBP1 target genes, also E2F7 has been demonstrated to be positively regulated. The combined activity of E2F7 and activated ATF6 will then bind the E2F1 promoter, resulting in specific E2F1 gene repression. The active repression of E2F1 thus requires a well-orchestrated, coordinated, and time-dependent process requiring both gene expression (E2F7) and protein activation (ATF6). The result is therefore a timely downregulation of E2F1 expression, this playing a key role in the survival/death transition as supported by the following evidence: (i) abrogated expression of the gene drastically accelerates the induction of the cell death program (thus inhibiting or bypassing the pro-survival ER stress branch), (ii) cells can revert the ER stress-mediated apoptotic program while the expression levels of E2F1 are still above a “threshold” (Figure 4). It is therefore possible to delineate a specific program set in motion by E2F1 downregulation by analyzing the downstream genes directly and indirectly regulated by this transcription factor. In fact, it specifically and negatively regulates the basal expression of the two main pro-apoptotic factors activated under ER stress condition, PUMA (DDIT3) and NOXA. Therefore, E2F1’s late downregulation re-establishes the levels of these pro-apoptotic factors that are early upregulated by ATF4, but whose activity is blocked by early/physiological expression of anti-apoptotic factors such as MCL-1 and BCL-2, respectively, and whose expression naturally declines quickly due to the decrease in ATF4 levels during the late phase of ER stress. Although the inhibition of MCL-1 expression is a direct consequence of ATF4 inactivation (27), BCL-2’s transcriptional repression results from GADD153/CHOP activity which, free from its negative regulator (TRB3) (28), might contribute to the induction of the pro-death pathway.

Taken together, all these evidence indicate that finely and timely regulated and coordinated expression levels of E2F1 are crucial for determining the survival/death cell fate under ER stress conditions (Figure 4).

## The ER Stress–Autophagy Connection: A Double-Edged Sword?

Although the UPR program is activated early under stress conditions and, depending on stimulus and duration, can trigger either survival or death, its close with another pro-survival cell program, such as autophagy, is becoming increasingly evident. In fact, several molecular pathways through which the UPR program can positively stimulate autophagy induction have been already described (29–31). On the other hand, it is also evident that autophagy may reversely inhibit the extension/duration of ER stress, by actively removing excessive ER membranes (ER-phagy) and including molecular components (structural and functional proteins) (32, 33). Autophagy is a physiological process by means of which double-membrane structures, mainly arising from the ER compartment, enwrap cytosolic proteins and organelles to form a vesicle named autophagosome. These vesicles are subsequently delivered to lysosomes, with the cargo digested after their fusion. This is a finely regulated and evolutionary well-conserved process controlled by specific ATG genes and proteins (34–36). A multiprotein complex formed by BECN1, AMBRA1, and ATG14 drives the formation of the autophagosomal membrane precursor (phagophore), by stimulating the class III PI3K VPS34. Next, several ATG proteins control the expansion and the closure of the nascent autophagosome with LC3 (the ortholog of the yeast ATG8) required for both expansion and the closure of the autophagosome. The subsequent fusion of autophagosomes with lysosomes results in cargo degradation and cytosolic release of building blocks that can be re-used by cells at occurrence. A large series of stress kinases regulates autophagy by targeting different components of the autophagy machinery, such as JNK and DAPK that positively regulates BECN1 by releasing its inhibitory interaction with the anti-apoptotic members of BCL-2 family (37).

The process can actively and specifically remove unwanted and/or damaged proteins and organelles in order to sustain cell survival under nutrient deprivation, hypoxia, chemical insults, and other conditions. Once digested, molecules are released into the cytosol as a pool of building blocks cells can use at their convenience.

**Figure 5:**
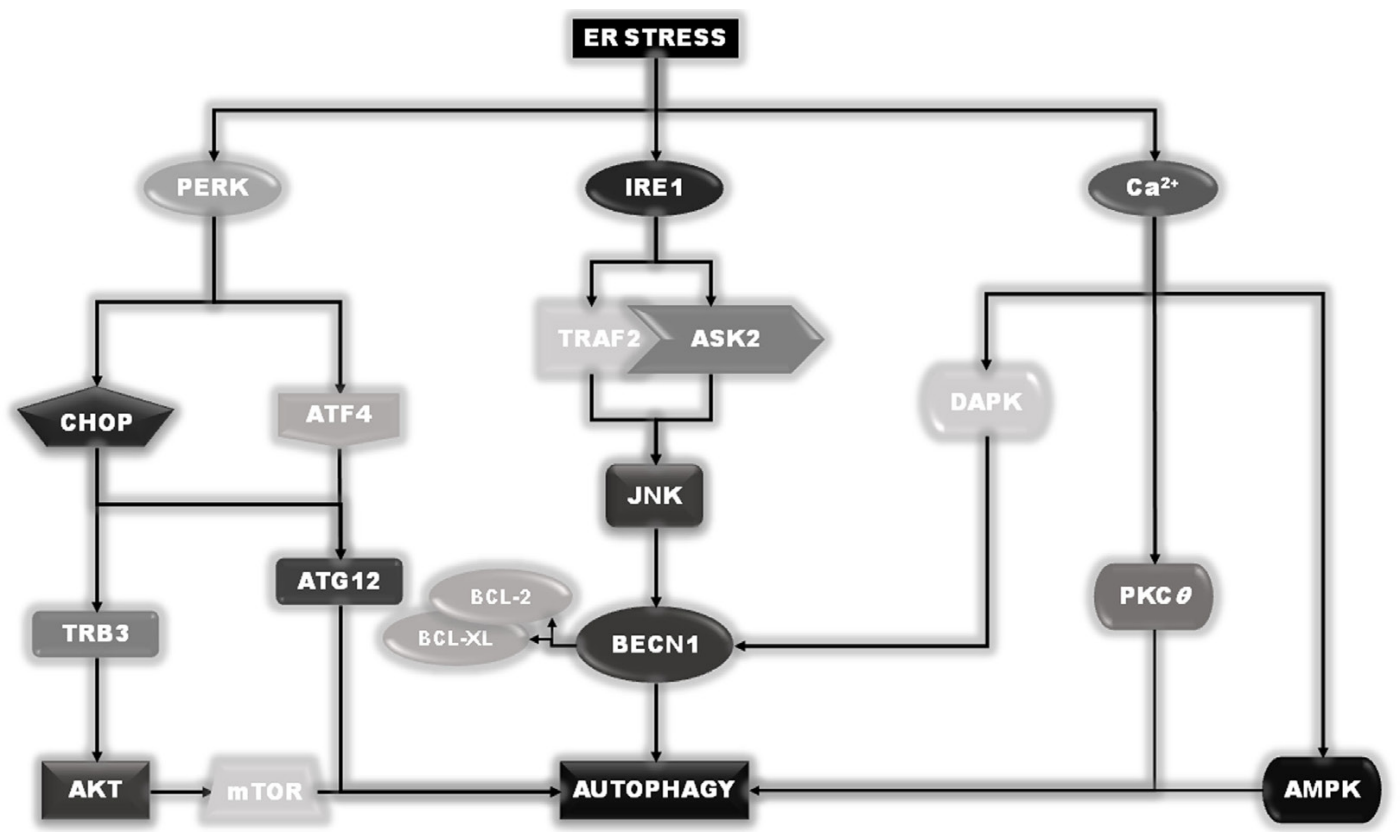
**Endoplasmic reticulum (ER) stress–autophagy connection**. Besides their role in the unfolded protein response program, pancreatic endoplasmic reticulum kinase and IRE1 are also involved in the ER stress-induced autophagy induction, acting thorough a direct stimulation of Beclin1 or the upstream autophagy regulator mTOR. Moreover, although the release of calcium is responsible for the induction of autophagy through the direct/indirect activation of DAPK, PKCθ, or AMPK, under ER stress conditions.

Under ER stress conditions, several signals emanate from ER to stimulate autophagy, some of them also initiated by the sensors IRE1 and PERK (29). In fact, as described earlier, active IRE1 can recruit TRAF2 and ASK2 to activate JNK. In turn, active JNK can phosphorylate the two autophagy inhibitory proteins, BCL-2 and BCL-XL, which dissociate from the key autophagy inducer BECN1 (31). Once released, BECN1 can stimulate the induction of the autophagic process. In addition, the activation of PERK will result in downstream expression of both ATF4 and GADD153/CHOP. ATF4 will drive the expression of ATG12. In combination with CHOP, it will positively regulate the expression of TRB3 (Tribbles Homolog 3) which, by inhibiting the AKT activity, will result in downstream inhibition of mTOR complex, further stimulating autophagy. Finally, calcium release from ER compartment can directly or indirectly stimulate the activity of enzymes such as DAPK, PKCθ, or AMPK, which positively stimulate the induction of the autophagic process (29) (Figure 5).

Upon induction, autophagy cooperates with UPR to sustain cell survival. When UPR is able to re-establish the physiological ER homeostasis, the ER-stimulated autophagy can therefore digest the excessive ER membranes and luminal proteins/enzymes, to re-establish physiologic ER size. Therefore, another question arising is: “How will ER stress-induced pro-survival autophagy be terminated to induce cell death?” Although this is not the subject of this dissertation and conclusive data are still missing, we can speculate that the same signaling responsible for autophagy inhibition under generic pro-apoptotic stimuli is still operative. In short, accumulation of active pro-apoptotic factors such as caspases may inhibit autophagy by active cleavage and inactivation of key autophagic proteins such as ATG5, ATG16A, BECN1, and AMBRA1 (37–39).

However, another point must be taken into account when discussing ER stress–autophagy connections and cell fate: autophagy can also stimulate apoptosis under ER stress conditions. In fact, although the protective role played by autophagy under ER stress conditions are well documented and widely accepted (as extensively discussed above), its role in participating in cell apoptosis under ER stress conditions is emerging only recently, is still poorly understood and highly debated. However, accumulating evidences indicate that autophagosome membranes might represent a platform for an intracellular death-inducing signaling complex-mediated caspase-8 activation, thus resulting in apoptosis initiation. In fact, inhibition of the early steps of autophagy activation resulted in the inhibition of caspase-8-mediated cell death (40, 41). Moreover, autophagy can also actively degrade anti-apoptotic factors such as inhibitor of apoptosis IAPs members. In fact, it has been recently found that PERK is implicated in the degradation of the anti-apoptotic XIAP (42). Finally, it emerged that cannabinoid (THC) treatment of melanoma cells resulted in the synthesis of ceramide, leading to ER stress stimulation and autophagic cell death induction. Importantly, autophagy inhibition prevented THC-induced autophagy and consequent cell death (43).

Altogether, these evidences indicate that the two processes ER stress and autophagy are intimately connected under cell stress conditions and their cooperation can result in both survival and death induction. However, further studies are required to clarify the conditions (cell/tissue type and stimuli) controlling and regulating this active cross talk and its outcome.

## ER Stress and Cancer

Cancer development is invariably characterized by uncontrolled growth and proliferation of transformed cells, resulting in a compact mass of cells, a tumor environment characterized by oxygen and glucose shortage, at least in solid tumors, two conditions that are canonical and well-characterized ER stress stimuli. Therefore, it is not surprising that UPR activation represents a hallmark of several human cancers, together with the upregulation of the ER stress master protein GRP78. In fact, UPR activation enables cancer cells to survive, adapts to adverse environmental conditions, and leads to growth arrest driving dormancy, which promotes resistance to conventional chemotherapy (44–47). Importantly, UPR is also involved in tumor-stimulated angiogenesis, particularly during the early exponential cell proliferation, when hypoxia and glucose shortage might compromise tumor growth. In fact, although UPR and hypoxia can independently induce angiogenesis by stimulating the expression of the master gene VEGF, through the PERK/ATF4 and the HIF1/2 pathways, respectively, the concomitant activation of the two signaling pathways results in impressive upregulation of VEGF, thus strongly stimulating angiogenesis, that is not merely the sum of the two independent stimulations (by ATF4 and HIF). In fact, this phenomenon is the result of both positive regulation of VEGF promoter by the two transcription factors and the stabilization of VEGF mRNA, thus resulting, altogether, in consistently enhanced gene expression (48, 49).

One example sustaining the role of UPR in tumorigenesis is represented by melanomagenesis. Indeed, although NRAS or BRAF mutations represent the main force driving melanoma development, they are not “*per se*” sufficient since these mutations are also present in benign nevi, thereby highlighting the requirement of other factors to drive melanocyte transformation and melanoma development (50, 51). Recent evidence has indicated that ER stress constitutes a key secondary event in melanoma development, contributing to resistance to apoptosis through the persistent expression of pro-survival instead of pro-apoptotic proteins (52). Moreover, in this context, UPR induction also results in basal autophagy enhancement, sustaining tumor cell survival, tumor growth, and resistance to chemotherapy (30, 31, 53). However, accumulating evidence indicates that the three branches of UPR can be differentially implicated in different tumor types and, interestingly, also in various tumor “phases,” such as development, progression, and resistance to therapy. In fact, a few of the well-characterized examples, supporting this notion, are that IRE1 signaling seems to be crucial during hepatocellular carcinoma (HCC) initiation, while PERK activation is required once the tumor had been established (54); PERK signaling is a critical factor in the adaptation of cancer cells to hypoxic stress in colorectal carcinoma (55), while promoting tumor dormancy under adverse microenvironmental conditions in squamous cell carcinoma (56); both UPR branches responsible for GRP78 upregulation and XBP1 production have been also implicated in tumor cells’ response to glucose deprivation, sustaining tumor cell survival (57, 58). However, all three UPR signaling factors have been recently observed coactivated and simultaneously involved in prostate cancer’s malignant progression (59).

Importantly, although the contribution of the ER stress response UPR to tumorigenesis has been associated with its “adaptive” main feature, conferring to cancer cells the ability to cope with stress thus resulting in tumor growth, progression, and resistance to therapy, another “dark side” of UPR is emerging and relying on mutations occurring in the three sensor genes: *ATF6, IRE1*, and *PERK*, in cancers. In fact, accumulating data are unveiling the presence of missense, nonsense, and silent mutations in these genes with the type of mutation apparently restricted to individual gene: missense mutations enriched in *PERK*, nonsense mutations enriched in *ATF6*, and silent mutations enriched in *IRE1*. However, the consequences of these mutations are only initiated to be elucidated, and further analyses are required to fully understand their impact on cell transformation and tumor development (60). Importantly, these mutations seem to have tumor/tissue specific significance, since mutational rates differ among cancers (61).

**Figure 6:**
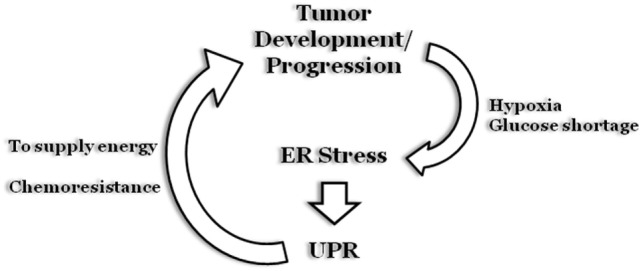
**Endoplasmic reticulum (ER) stress and cancer**. During early stages of solid tumors development, uncontrolled cell proliferation results in the formation of a “cell core” characterized by hypoxia and glucose shortage. The two stress stimuli promptly stimulate an ER stress status and a subsequent unfolded protein response (UPR) program that will sustain tumor cell survival. Moreover, the UPR program can also be stimulated as a result of chemotherapy treatment, to inhibit cell death induction, thus sustaining tumor growth.

Therefore, the emerging scenario indicates that, although UPR is involved in all stages and/or phases of tumorigenesis, the role and impact of each component tend to be tumor specific, and further studies are required to fully decipher the real impact of UPR on cancer (Figure 6).

## Targeting ER Stress as a Therapeutic Strategy

Since ER homeostasis together with the expression of many ER proteins, such as GRP78, CLX (calnexin), and ERp57, are frequently altered and dysregulated in many cancer types such as neuroectodermal and hepatic cancers, conferring cell growth advantages and resistance to death, this compartment could be conceivably considered a potential target for cancer therapies. In fact, it has been previously reported that ER stress-associated markers are specifically upregulated in both neuroblastoma and melanoma cells under ER stress conditions (20). A deeper analysis of UPR in these cells highlighted an upregulation of several members of the PDI family. Briefly, this is a group of at least 20 proteins sharing a dithiol-disulfide oxidoreductase and molecular chaperone activity, responsible for disulfide bond oxidation (formation), reduction (breakage), and isomerization (rearrangement) of peptides and proteins, thus mediating oxidative protein folding. Moreover, they bind and stabilizes the major histocompatibility complex (MHC) class I peptide-loading complex that mediates MHC class I folding and peptide loading (62). Their dysregulated activity and/or expression has been implicated in tumor progression, angiogenesis, invasion, and in resistance to therapy (63). In fact, ERp57 (PDIA3) and ERdj5 (PDIA19) were consistently upregulated in both neuroectodermal tumors, with the abrogation of their expression, resulting in enhanced cell death induction under ER stress conditions. Importantly, dysregulation of both expression and activity of PDI is also associated with pathological conditions beyond cancer, such as neurodegenerative and cardiovascular diseases (64, 65). Moreover, a generalized inhibition of PDI activity revealed a significant sensitization of both neuroectodermal tumor cells to ER stress-mediated apoptosis induction, supporting the crucial role played by these enzymes under ER stress conditions (20, 66). Importantly, although the exact role(s) played by PDI in cancer progression is still not well established, PDI inhibition appears as a fascinating novel strategy for sensitizing different cancer types beyond neuroectodermal malignancies to apoptosis, such as multiple myeloma and HCC (67, 68). However, is also important to note that despite the intense study in last decades, there are still no selective PDI inhibitors for clinical use. Recently, two synthetic small-molecule inhibitors, PACMA 31 and 16F16, have proven efficacy in cancer models and although further evaluation of their specificity and off-target effects is needed, they can help to select viable candidates for clinical studies (63).

GRP78, ERp57, and ERdj5 have been also found to be dysregulated in many other tumors, while their specific targeting resulted in enhancing tumor cells’ capacity to respond to therapy, possibly resulting in increased overall clearance of tumors (20, 69–71).

Importantly, GRP78 downregulation/knockdown resulted in (i) inhibited ability of fibrosarcoma cells to form tumors upon xenographting into mice, (ii) decreased breast adenocarcinoma growth in a mouse model, and (iii) decreased growth rate in glioma cells (72–74). Moreover, altered expression of these proteins has been also observed in other tumors such as melanoma, lung, head and neck, brain, breast, prostate, and HCC (47, 75–77). This supports the strong involvement of this factor in cancer development, progression, and resistance to therapy, along with its candidature as therapeutic target.

On the other hand, generalized chronic ER stress has been observed in many tumors and is constantly used to sustain cell survival and resistance to therapies. This phenomenon is of particular interest in human skin melanomas characterized by oncogenic BRAF mutations, such as BRAF^V600E^. Interestingly, the expression of ER stress markers compatible with a chronic condition has been revealed not only in oncogenic BRAF melanoma cell lines but also in patients who failed the clinical treatment. In both models, the presence of oncogenic BRAF was strictly responsible for ER stress induction and cell survival (30, 31, 78). Moreover, BRAF-dependent chronic ER stress was also linked to basal autophagy enhancement responsible for resistance to therapy in cooperation with UPR program. Therefore, the sole inhibition of autophagy failed to sensitize tumor cells to apoptosis while ER stress buffering, by using a genetic approach or chemical chaperones, efficiently decreased basal autophagy and effectively re-sensitized cells to apoptosis induction, so highlighting the pivotal role played by the UPR program in controlling both autophagy and cell resistance to therapy (30, 78).

Finally, one can speculate that those elements particularly important in the survival/death switch control may represent specific tumor therapeutic targets. This is particularly true for E2F1, since its deregulated expression in several tumor types, including breast cancer, is associated with enhanced resistance to therapy, in accordance with the pivotal role played by this transcription factor in the control of survival/death cell decisions under stress conditions. Therefore, pharmacological inhibition of E2F1 expression may significantly enhance cancer sensitivity to pro-death agents reducing/abrogating the pro-survival branch of UPR program.

Pro-survival ER stress activation has been also demonstrated in cancer stem cells (CSC), responsible for tumor regeneration. Importantly, a pivotal role of ER stress has been demonstrated in CSC resistance to therapy, with different branches of UPR program apparently activated and responsible for cell survival depending on the stress stimulus applied. However, specific inhibition of the three sensors coupled to pro-death agent administration consistently enhanced CSC sensitivity to therapy (79).

Although targeting the UPR effectors is a relatively novel strategy with potential clinical implications, some interesting clinical and pre-clinical trials have started with available data indicating that this could represent one of the best therapeutic strategies to treat multiple cancer types (49).

## Conclusion

Accumulating data indicate and support the concept that the UPR program represents a key process in tumor development, stage progression, and resistance to therapies. However, it is now becoming evident that the “dark side” of the process, represented by the pro-death branch, might be used to successfully treat such malignancies and to overcome their intrinsic or acquired resistance to therapy. In this scenario, it has become crucial to better understand the event(s) regulating the survival/death switch under ER stress conditions. Moreover, the question “to stimulate or inhibit” UPR to increase cancer cell sensitivity to death is still a challenge since a deeper knowledge of each tumor must be taken into account, to show the path. Moreover, it is important to consider that there are still no specific and clinically available “modulators,” either positive or negative, of UPR. Therefore, an intensive effort is still required to (i) better define the role of UPR in specific tumors, (ii) unveil the role of each UPR branches in each tumor, and (iii) identify/develop drugs with high target specificity and low side effects. However, in our opinion, the deeper description at molecular level of the “survival/death switch” under ER stress conditions remains the key point. Therefore, the specific pharmacological modulation of this switch might represent the future goal of this field of research.

## Author Contributions

MC conceived and wrote the manuscript; MP and GF provided editorial advice; MG produced images and involved in manuscript editing.

## Conflict of Interest Statement

The authors declare that the research was conducted in the absence of any commercial or financial relationships that could be construed as a potential conflict of interest.
